# Ultra-rapid cooling of ibex sperm by spheres method does not induce a vitreous extracellular state and increases the membrane damages

**DOI:** 10.1371/journal.pone.0227946

**Published:** 2020-01-24

**Authors:** Paula Bóveda, Adolfo Toledano-Díaz, Cristina Castaño, Milagros Cristina Esteso, Antonio López-Sebastián, Dimitrios Rizos, Alejandro Bielli, Rodolfo Ungerfeld, Julián Santiago-Moreno

**Affiliations:** 1 Dpto. Reproducción Animal, INIA, Madrid, Spain; 2 Dpto. Morfología y Desarrollo, Facultad de Veterinaria, Universidad de la República, Montevideo, Uruguay; 3 Dpto. Fisiología, Facultad de Veterinaria, Universidad de la República, Montevideo, Uruguay; Peking University Third Hospital, CHINA

## Abstract

Sperm cryopreservation by ultra-rapid cooling based on dropping small volumes of sperm suspension directly into liquid nitrogen, has been successful in some wild ruminant species, including the Iberian ibex (*Capra pyrenaica*). In ultra-rapid cooling, the contents of these droplets are expected to enter a stable, glass-like state, but to the best of our knowledge no information exists regarding the presence or absence of ice formation in the extracellular milieu when using this technique. Different modifications to the extracellular milieu likely inflict different types of damage on the plasmalemma, the acrosome and mitochondrial membranes. The aims of the present work were: 1) to examine the physical state of the extracellular milieu after cryopreservation at slow and ultra-rapid cooling rates—and thus determine whether ultra-rapid cooling vitrifies the extracellular milieu; and 2) to compare, using conventional sperm analysis techniques and scanning and transmission electron microscopy, the damage to sperm caused by these two methods. Sperm samples were obtained by the transrectal ultrasound-guided massage method (TUMASG) from anesthetized Iberian ibexes, and cryopreserved using slow and ultra-rapid cooling techniques. Sperm motility (22.95 ± 3.22% vs 4.42 ± 0.86%), viability (25.64 ± 3.71% vs 12.8 ± 2.50%), acrosome integrity (41.45± 3.73% vs 27.00 ± 1.84%) and mitochondrial membrane integrity (16.52 ± 3.75% vs 4.00 ± 0.65%) were better after slow cooling (P<0.001) than after ultra-rapid technique. Cryo-scanning electron microscopy (Cryo-SEM) suggested that the vitrified state was not achieved by ultra-rapid cooling, and that the ice crystals formed were smaller and had more stretchmarks (P<0.001) than after slow cooling. Scanning electron microscopy revealed no differences in the types of damage caused by the examined techniques, although transmission electron microscopy showed the damage to the plasmalemma and mitochondrial membrane to be worse after ultra-rapid cooling. In conclusion ultra-rapid cooling provoked more membrane damage than slow cooling, perhaps due to the extracellular ice crystals formed.

## Introduction

Much effort has been invested in improving and simplifying the techniques for cryopreserving the sperm of wild ungulates and thus facilitate their use under field conditions [1]. Iberian ibex is a mountain caprine endemic to the Iberian Peninsula that display marked vulnerability to environmental and sanitary factors. It has a short breeding season that extends from December to February, although spermatogenic activity remains through the year with the lowest sperm quality in spring [2]. The successful use of assisted reproduction technologies with this species is a useful model for *ex situ* conservation strategies designed to preserve other threatened wild ruminants [3]. Recent studies have reported live offspring of ibexes from artificial insemination with frozen and vitrified sperm [4, 5, 6].

Cryoinjury during conventional freeze-thawing is caused by factors such as thermal shock, ice formation, dehydration, increased salt concentration and osmotic shock [7, 8]. The cooling rate affects the extracellular ice formation; the cells and the dissolved salts are excluded from the ice and become concentrated in the unfrozen fraction remaining between the growing ice masses [9]. Thus, the osmotic strength of the unfrozen fraction increases, causing an efflux of water from the cells, resulting in cell shrinkage [10]. While conventional freezing requires the use of cryoprotectants to prevent damage caused by ice crystal formation, vitrification techniques allow glass transition and forms and stable structure without the presence of ice crystals through high concentrations of permeable cryoprotective agents [11] and/or very rapid freezing rates (kinetic vitrification) [12]. Sperm vitrification was first developed for use with humans as a means of simplifying and speeding up cryopreservation without the need for sophisticated equipment, making it cheaper than traditional protocols [12, 13]. High concentrations of cryoprotectant are harmful to sperm cells [5]; sperm vitrification, however, involves ultra-rapid cooling of small-volume samples without permeable cryoprotectant [14]. Rapid cooling rates should prevent formation of ice crystals inside the cells, and thus, the entire cell suspension should vitrify [15]. Some non-permeable additives with cryoprotective activity, such as human serum albumin and sucrose, have been successfully used in the kinetic vitrification of human sperm [16]. This modified method was subsequently used to successfully cryopreserve dog [17] and fish sperm [18], and more recently Iberian ibex (*Capra pyrenaica*) [6] and European mouflon (*Ovis musimon*) [19] sperm. Vitrification occurs—or not—depending on the cooling rate, the viscosity of the solution, and the volume to be preserved [20]. However, the term ‘vitrification’, should be used only when both the intracellular milieu and the extracellular environment of the sperm cells become vitrified [16]. The most common ultra-rapid cooling technique is based on dropping small volumes of sperm suspension directly into liquid nitrogen [6, 21, 22]. To the best of our knowledge, however, no information is available regarding the formation—or not—of ice in the extracellular milieu when using this procedure. Extracellular ice is a primary cause of sperm damage during the cryopreservation process [23].

The plasmalemma and acrosomal membranes are major sites of cryopreservation-induced sperm damage [24]; this is certainly true for wild ruminant sperm vitrified in pellets [19]. The size of the sperm head can also be affected. Although in most of the species studied, sperm cells are made smaller by conventional freezing-thawing [25, 26, 27], a recent study that included observations on several wild species reported that cryopreservation at very high cooling rates may lead to an increased sperm head size after freezing-thawing [28]. Motility has also been reported lower in frozen-thawed vitrified sperm than in conventionally cryopreserved sperm [19]. Reduced mitochondrial activity usually underlies reduced kinetic activity [29], which is consistent with the reduced mitochondrial functionality seen in vitrified human sperm after thawing [21]. Alterations to the mitochondrial membrane, and ultrastructural damage to the axoneme, can be caused by intracellular and extracellular ice produced during the freezing process, with the severity of the damages perhaps differing depending on the cryopreservation method used [30, 31].

The aims of the present study were therefore: 1) to determine whether the extracellular milieu of Iberian ibex sperm samples is vitrified during a specific ultra-rapid cooling protocol previously used in this species; and 2) to compare the damage caused to the species' sperm when cryopreserved by ultra-rapid cooling and slow cooling. Data were compared with these obtained using slow freezing by cryopreservation methods previously tested in ibexes. We used the more successfully procedure [32], which varied in cryoprotectants and even number of sperm per sample (straw) in comparison with ultra-rapid procedure in pellets.

## Material and methods

### Animals and sperm collection

Animals were handled according to procedures approved by the INIA Ethics Committee (Órgano Regulador de los Comités de Ética de Experimentación Animal) that specifically approved the design of the current study (reference number ORCEEA 2014/027; reference regional government PROEX 271/14) and were performed in accordance with the Spanish Policy for Animal Protection (RD53/2013), which conforms to European Union Directive 86/609 regarding the protection of animals used in scientific experiments. Iberian ibexes were housed in captivity at the INIA Department of Animal Reproduction. All had born at the INIA facilities. They were kept in a 250-m^2^ enclosure with partial roof cover. All were fed Visan K-59 (Visan Ind. Zoot. S.A, Madrid, Spain) containing 15% crude protein, 15.7% crude fibre, 4% crude fat, 10.6% crude ash, 0.5% Na. This commercial feed was supplemented with barley grain, barley straw, and dry alfalfa. Water and vitamin/mineral blocks were available *ad libitum*. To alleviate stress during experimental procedures, animals were accustomed to handling in a small restraining stall (2-m^2^) in which anesthesia was administered. The animals were anesthetized using 50 μg/kg intravenous detomidine (Domosedan®) (Pfizer Inc., Amboise Cedex, France), 0.5 mg/kg ketamine hydrochloride (Imalgene-1000®) (Rhône Mérieux, Lyon, France) and 0.5 mg/kg tiletamine-zolazepam (Zoletil-100®) (Virbac España S.A., Barcelona, Spain). Anesthesia was maintained with 1.5% isoflurane (Isobavet®) (Intervet/Schering Plough Animal Health, Madrid, Spain) in oxygen (flow rate 2.5 L/min) administered via an endotracheal tube. Pulse oximetry and capnography were used to monitor the condition of the animals. During all manipulations the eyes were covered with a mask to further reduce stress.

Fourteen sperm samples were collected by transrectal ultrasound-guided massage of the accessory glands (TUMASG) [33] from six anesthetized Iberian ibexes. Briefly, ultrasound examination of the bulbourethral glands, the seminal vesicles, and the ampulla of the vas deferens was performed using real-time transrectal ultrasonography employing a 7.5 MHz linear array probe (Prosound 2, Aloka CO., LTD, Tokyo, 181–8622 Japan). TUMASG was performed with the ultrasonographic probe placed on the ampulla of the vas deferens, using a back-and-forth motion to favour the expulsion of the spermatozoa. If the animal did not ejaculate, electrical stimuli (0.2 mA lasting 6–8 s) were provided with an electroejaculator, with intermittent breaks for TUMASG; a maximum of 1–3 electrical stimuli were usually required. The electroejaculator used was a Lane Pulsator IIIZ model (Lane Manufacturing Inc., Denver, Colorado, USA) consisting of a rectal probe 2.5 cm in diameter and 20.5 cm in length. The process was monitored by ultrasound scanning of the ampulla of the vas deferens, verifying that the emptying of the glands was complete. Stimulation was halted when the echogenicity of the ampulla was compatible with its being empty.

The diluents and all materials coming into contact with the semen were maintained at 37° C. The volume of the ejaculates was measured using a micropipette (Gilson, Villiers Le Bel, France). The percentage of motile sperm and the quality of motility were initially evaluated via phase contrast microscope (Zeiss, Oberkochen, Germany). Only those ejaculates with a sperm motility value of >50%, and a score of >2 on a motility scale of 0 (lowest) to 5 (highest), were used in the subsequent experimental work. The samples were separated into two fractions to be diluted with one of two experimental extenders prepared in-house (using reagent-grade chemicals purchased from Panreac Química S.A. [Barcelona, Spain] or the Sigma Chemical Co. [St. Louis, Missouri, USA] depending on the freezing method used) until reaching a final concentration of 100 x 10^6^ sperm/mL. The extender used for the conventional slow cooling samples was TCG (3.8% Tris [wt/vol], 2.2% citric acid [wt/vol], 0.6% glucose [wt/vol] + 6% egg yolk [vol/vol], plus glycerol at 5% [vol/vol]; osmolality: 1150 mOsm/kg). The extender for the ultra-rapid cooling samples was the same, with the glycerol substituted by 100 mM sucrose (osmolality: 486 mOsm/kg). The osmolality measured in the absence of cryoprotectants of the TCG extenders was 345 mOsm/kg. All solutions were adjusted to pH 6.8, with NaOH at room temperature.

### Sperm cryopreservation

The sperm samples cryopreserved by ultra-rapid cooling were cooled for 30 min at 5°C and then plunged drop-by-drop (about 50 μL/drop) directly into liquid nitrogen as reported by Pradiee et al. [5]. Sperm samples cryopreserved by conventional slow cooling were cooled at 5ºC for 1 h, and then maintained at this temperature for 2 h (total equilibration therefore 3 h). Aliquots were then loaded into 0.25 mL straws and frozen by placing them in nitrogen vapour 5 cm above the surface of a liquid nitrogen bath for 10 min [32]. Twelve months later the above cryopreserved sperm pellets were thawed by placing them on a DPP70 thermo-regulated conical hotplate (INIA, Madrid, Spain) set at 60–65°C, and the straws thawed in a water bath at 37°C for 30 s. Previous reports in our lab showed fast warming to be important in preventing damage to ibex sperm cryopreserved at high cooling rates [5].

### Conventional sperm analysis by fluorescence and the computer-aided sperm analysis (CASA) system

Sperm variables were assessed before and after cryopreservation. Total sperm concentration was determined in fresh semen using a Neubauer chamber (Marienfeld, Lauda-Königshofen, Germany). The total percentage of motile sperm and the straight line velocity (VSL) were determined using a computer-aided sperm analysis system (CASA) (SCA, Barcelona, Spain) coupled to a Nikon Eclipse model 50i phase contrast microscope with negative contrast capacity [33]. A minimum of three fields and 500 sperm were assessed.

Morphometric analyses were made on 10 samples (fresh and frozen-thawed for each cryopreservation method). Smears were stained with Hemacolor® (Merck KGaA, Darmstadt, Germany) and assessed using the ISASv1 system (Projectes I Serveis R+D, Valencia, Spain). The sperm head dimensions length (L), width (W), area (A), and perimeter (P), were determined by examining 100 sperm cell heads [34].

The status of the plasma membrane, acrosome membrane and mitochondrial membrane was assessed in 10 frozen-thawed samples cryopreserved by each method, using an association of propidium iodide (PI, Sigma P4170), fluorescein isothiocyanate-conjugated peanut (*Arachis hypogaea*) agglutinin (PNA-FITC, Sigma L7381) and Mitotracker Green FM (MITO, Invitrogen M7514, USA) following the method of Forero-Gonzalez et al. [35] with slight modifications. An aliquot of 150 μL of semen diluted in TALP stock medium at 25 x 10^6^ sperm/mL was mixed with 2 μL of PI (2 mg/mL), 1 μL of MITO (0.5 mM) and 50 μL of FITC-PNA,100 μg/mL) and incubated in the dark at 38.5°C for 8 min. Live sperm were classified into eight categories according to their plasmalemma, acrosomal and mitochondrial staining: 1) sperm with an intact plasmalemma, an intact acrosome and an intact mitochondrial membrane (IP-IA-IM); 2) sperm with an intact plasmalemma, an intact acrosome and a damaged mitochondrial membrane (IP-IA-DM); 3) sperm with an intact plasmalemma, a damaged acrosome and an intact mitochondrial membrane (IP-DA-IM); 4) sperm with an intact plasmalemma, a damaged acrosome and a damaged mitochondrial membrane (IP-DA-DM); 5) sperm with a damaged plasmalemma, an intact acrosome and an intact mitochondrial membrane (DP-IA-IM); 6) sperm with a damaged plasmalemma, an intact acrosome and a damaged mitochondrial membrane (DP-IA-DM); 7) sperm with a damaged plasmalemma, a damaged acrosome and intact mitochondrial membrane DP-DA-IM); 8) sperm with a damaged plasmalemma, a damaged acrosome and a damaged mitochondrial membrane (DP-DA-DM). A total of 200 sperm cells per sample were analyzed.

### *Cryo-scanning electron* microscopy *(Cryo-SEM)*

The Cryo-SEM was made in the Universitat Politècnica De València. The extracellular milieu of samples cryopreserved by the slow cooling (three samples [in straws]) and ultra-rapid cooling (three samples [in pellets]) methods was studied by Cryo-SEM. Cryopreserved samples of each type, containing about 25 million of sperm per straw and 5 million of sperm per pellet, maintained at all times in liquid nitrogen, were manually fractured; the straw samples were broken into sections 0.3–0.5 cm long, while the pellets were broken in half. All samples were then mounted on a mechanical cryo-transfer holder, maintaining them at -196ºC, and fixed with TBS Tissue Freezing Medium for frozen tissue specimens (Triangle Biomedical Sciences, Durham, NC, USA), which solidified in contact with the liquid nitrogen. The grip holder was transferred into a chamber containing snow nitrogen, and then moved into an Oxford CT1500 cryostage chamber (Oxford, UK) attached to a JEOL JSM 5410 Scanning Microscope (Japan). The temperature of sample is raised by heating the holder to -90ºC for 5–10 min in order to sublimate free water in the solid state lakes, followed by a temperature decrease to -130ºC to stabilize the sample. The coated sample was then transferred to the microscope chamber where it was analyzed at a temperature range of -125 to -135ºC. The glass transition temperature is raised over -90º C in hypertonic solutions (e.g. sucrose-egg yolk based extender for the ultra-rapid cooling), and thus the frozen state was not affected in the sublimation step [9, 36]. The frozen preparation was then gold-sputtered. Digital images were obtained at 15 kV using an Oxford CT500 camera running INCA Oxford software (Oxford, UK), at magnifications of 750–3500. As a control, straws containing a conventional extender for vitrifying embryos (a final solution containing 20% ethylene glycol and 20% DMSO as cryoprotectants) [37] were analyzed to compare its frozen state with the test samples. Straw sections of 1 cm long were cut, placed on the cryo-transfer holder and analyzed as above. ImageJ v.1.8.0 software (National Institutes of Health (NIH), Maryland, USA) was used to determine the size of the crystals produced by each cooling method. The crystal perimeter and four crystal shape descriptors were also recorded: *circularity* (4π × [Area]/[Perimeter]^2^) (a value of 1.0 indicates a perfect circle; values approaching 0.0 indicate an increasingly elongated shape [values may not be valid for very small crystals]; *aspect ratio* of the crystals' fitted ellipse, i.e., [Major Axis]/[Minor Axis]; *roundness*, i.e., the inverse of the aspect ratio; and *solidity* ([Area][Convex area]).

### Scanning electron microscopy (SEM)

The SEM was made in the ICTS-Centro Nacional de Microscopía Electrónica from the Universidad Complutense of Madrid. Cryopreserved straws and pellets (four samples for each method) were thawed as described in sperm cryopreservation section, transferred to 750 μl of a fixative solution (4% paraformaldehyde and 2.5% glutaraldehyde), maintained for 4 h at 5°C, and filtered using fluoropore filters (13 mm diameter and pore size 0.2 μm) (Merck Millipore Ldt. Cork, Ireland). The sperms remaining on the filters were washed (twice) directly over using distilled H_2_O and then, filters were introduced in a test tube containing distilled water for 10 min. After dehydration through an ascending series of alcohol solutions, the material was critical point dried using a Leica EM CPD 300 critical point dryer (Leica, Wetzlar, Germany). Then, they were gold-sputtered and examined using a Jeol JSM 6400 electron microscope (Tokyo, Japan). Images were captured using INCA Oxford software (Oxford, UK) and the sperms examined for structural damage to the sperm head, acrosome membrane and midpiece.

### Transmission electron microscopy (TEM)

The TEM was made in the ICTS-Centro Nacional de Microscopía Electrónica from the Universidad Complutense of Madrid. Four frozen-thawed samples of sperm cryopreserved by the two methods were diluted 1:9 (vol/vol) with a wash medium composed of 3.8% Tris (wt/vol), 2.2% citric acid (wt/vol), 0.6% glucose (wt/vol), and centrifuged at 1.2 *g* for 15 min. The supernatant was then removed and the pellet resuspended and fixed in 2 ml of Karnovsky solution at 5°C for 4 h. This was followed by washing with Milloning buffer 1.2 g for 3 x 15 min, postfixing in 1% OsO_4_ for 1 h, and washing in miliQ water (3 x 10 min). The samples were then dehydrated in an ascending series of acetone plus SPURR resin (1:3 for 1 h, 1:1 for 1 h and 3:1) overnight at 65–70°C. Ultrathin sections were cut using a Reicher Ultracut S ultramicrotome (Leica, Wetzlar, Germany) and examined on copper grids with 200 hexagonal meshes using a JEM 1400 electron microscope (Tokyo, Japan) equipped with a Digital Micrograph image analysis system (Gatan, California, USA). Images of sperm cells were assessed qualitatively for changes in plasmalemma, mitochondrial and midpiece ultrastructure.

### Statistical analysis

Data are expressed as means ± SE. The normality of data distribution was examined using the Shapiro–Wilks test; non-normal values were arcsine-transformed before analysis. The paired t-test was used to compare the effect of cryopreservation method on the examined sperm variables within and between cryopreservation methods. A total of 14 sperm samples were analysed.

GLM nested ANOVA was used to compare cryopreservation methods in terms of the shape and size of the ice crystals produced, following the statistical model X_ij_ = m + T_i_+ A_j(i)_ + e_ijk_, where X_ij_ = accuracy of the assay, m = overall means of crystals variables, T_i_ = cryopreservation method (i = 1–2), A_j(i)_ = effect of the number of samples (j = 1–3 for T_1_ and j = 1–3 for T_2_), and e_ijk_ = residual (k = 1–100).

Where applicable, significance was set at p<0.05. All analyses were performed using STATISTICA software for Windows v.12.0 (StatSoft, Inc., Tulsa, OK, USA).

## Results

### Sperm evaluations by CASA and fluorescence microscopy

The fresh sperm samples had 78.43 ± 4.17% viable sperms, 94.71 ± 0.97% sperms with an integral acrosome, and 65.04 ± 5.16% motile sperms. The straight line velocity (VSL) of these sperms was 40.20 ± 4.10 μm/s. After freezing-thawing, the percentage of motile sperms and the VSL values were higher for the samples cryopreserved by slow cooling (22.95 ± 3.22% *vs* 4.42 ± 0.86% for those cryopreserved by ultra-rapid cooling, and 38.06 ± 3.45 μm/s *vs* 25.11 ± 3.96 μm/s for those cryopreserved by ultra-rapid cooling; P<0.001 and P<0.02 respectively). For both cryopreservation techniques, the sperm head dimensions were smaller after freezing-thawing compared to fresh sperm (P<0.05 for both methods) (Table 1).

**Table 1:** Morphometric values (mean ± SE) for fresh and thawed Iberian ibex sperm subjected to either slow conventional freezing or ultra-rapid cryopreservation.

	Fresh	Post conventional freezing	Post ultra-rapid freezing
Length (μm)	8.42 ± 0.06^a^	8.15 ± 0.08^b^	8.17 ± 0.07^b^
Width (μm)	4.27 ± 0.03^a^	4.30 ± 0.04^a^	4.25 ± 0.02^a^
Area (μm^2^)	29.71 ± 0.29^a^	28.60 ± 0.53^a^	28.59 ± 0.17^b^
Perimeter (μm)	22.48 ± 0.13^a^	21.97 ± 0.19^b^	21.98 ± 0.12^b^

Different lower case letters (a, b) indicate significant differences (P < 0.05) between fresh samples and post-thawing after each cryopreservation method.

**Fig 1:**
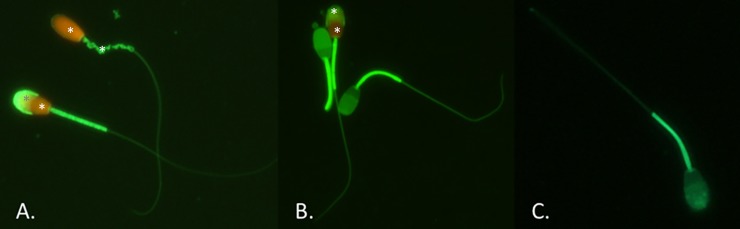
Sperm stained with an association of propidium iodide, fluorescein isothiocyanate-conjugated peanut agglutinin and Mitotracker Green FM (x400). A. Dead sperm cell with a damaged plasmalemma, an intact acrosome and a damaged mitochondrial membrane (DP-IA-DM, on top), and a dead sperm cell with a damaged plasmalemma, a damaged acrosome and an intact mitochondrial membrane (DP-DA-IM, on bottom). B. Two viable sperm cells with an intact plasmalemma, an intact acrosome and an intact mitochondrial membrane (IP-IA-IM), and one dead sperm cell (in the middle) with a damaged plasmalemma, a damaged acrosome and an intact mitochondrial membrane (DP-DA-IM). C. Viable sperm cell with an intact plasmalemma, an intact acrosome and an intact mitochondrial membrane (IP-IA-IM). Asterisks indicate the sperm damages.

The percentage of sperm with intact plasmalemma, intact acrosome and with intact mitochondrial membrane (IP-IA-IM category sperm) was greater after slow cooling than after ultra-rapid cooling (Table 2; P<0.01), while the percentage of sperm with damaged plasmalemma, damaged acrosome and damaged mitochondrial membrane (DP-DA-DM category sperm) was greater after ultra-rapid cooling than after slow cooling (Table 2; P<0.01). The total percentage of sperm with intact mitochondria, the total percentage with an intact plasma membrane, and the total percentage with an intact acrosome, was greater after slow cooling than after ultra-rapid cooling (P<0.05) (Table 2; Fig 1).

**Table 2:** Percentage of sperm with intact/damaged plasmalemma, acrosome, and mitochondrial membrane of Iberian ibex sperm subjected to either slow conventional freezing or ultra-rapid cryopreservation. Values are mean ± SE.

	Post slow freezing	Post ultra-rapid freezing
IP-IA-IM (%)	14.01 ± 2.83^a^	3.20 ± 0.57^b^
IP-IA-DM (%)	11.08 ± 2.04^a^	9.30 ± 1.92^a^
IP-DA-IM (%)	0.25 ± 0.13^a^	0.10 ± 0,10^a^
IP-DA-DM (%)	0.3 ± 0.21^a^	0.20 ± 0.20^a^
DP-IA-IM (%)	1.1 ± 0.69^a^	0.20 ± 0.13^a^
DP-IA-DM (%)	15.26 ± 2.20^a^	14.3 ± 2.26^a^
DP-DA-IM (%)	1.16 ± 0.67^a^	0.50 ± 0.16^a^
DP-DA-DM (%)	56.82 ± 4.15^b^	72.20 ± 1.80^a^
Total IM (%)	16.52 ± 3.75^a^	4 ± 0.65^b^
Total IP (%)	25.64 ± 3.71^a^	12.8 ± 2.50^b^
Total IA (%)	41.45 ± 3.73^a^	27 ± 1.84^b^

Different lower case letters (a, b) indicate significant differences between cryopreservation methods. IP-IA-IM: sperm with intact plasmalemma, intact acrosome and with intact mitochondrial membrane; IP-IA-DM: sperm with intact plasmalemma, intact acrosome and damaged mitochondrial membrane; IP-DA-IM: sperm with intact plasmalemma, damage acrosome and intact mitochondrial membrane; IP-DA-DM: sperm with intact plasmalemma, damaged acrosome and damaged mitochondrial membrane. DP-IA-IM: sperm with damaged plasmalemma, intact acrosome and with intact mitochondrial membrane; DP-IA-DM: sperm with damaged plasmalemma, intact acrosome and damaged mitochondrial membrane; DP-DA-IM: sperm with damaged plasmalemma, damaged acrosome and intact mitochondrial membrane; DP-DA-DM: sperm with damaged plasmalemma, damaged acrosome and damaged mitochondrial membrane. IM: total sperm with intact mitochondrial membrane; IP: total sperm with plasmalemma integrity; IA: total sperm with intact acrosome.

### Cryo-SEM

**Fig 2:**
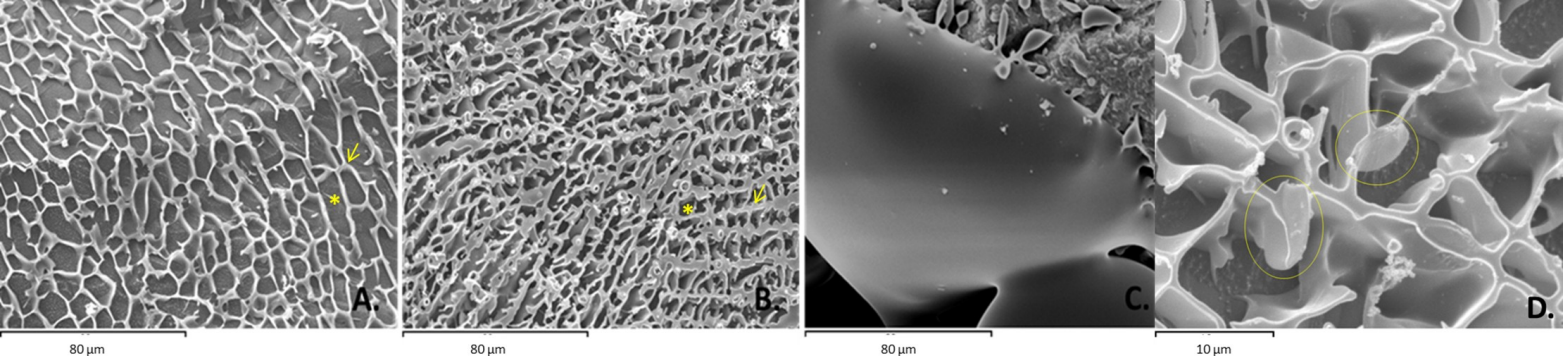
Cryo-SEM images of the distribution of ice crystals within the extender matrix (mag. x750). **A.** Cryopreservation state after slow cooling in straws with 5% glycerol as a cryoprotectant. **B.** Cryopreservation state after ultra-rapid cooling as pellets (50 μl) with 100 mM sucrose as a cryoprotectant. **C.** Cryopreservation state of a conventional medium used for embryo vitrification (control). Asterisks showed the lakes and arrows showed the veins. **D.** Two sperm heads (circles) entrapped in the freeze matrix, without evident intracellular ice formation.

The Cryo-SEM appearance of the frozen, slow-cooled and ultra-rapid cooled extenders differed from that of the frozen control extender used to vitrify embryos. The latter appeared totally homogeneous, while, both the frozen test extenders showed ‘lakes’ (small areas occupied by pure ice) and veins, i.e., areas with high solute concentrations (Fig 2). Thus, Cryo-SEM revealed ultra-rapid cooling not to induce a stable, glass-like extracellular state. Nevertheless, after ultra-rapid cooling, the crystals were smaller (P<0.001 for both perimeter and area aspect ratio) and more stretchmarked than those observed after slow cooling. The circularity and roundness values of the ultra-rapid cooling-associated crystals were higher (P<0.001 for each variable). However, no differences were seen in the solidity of the crystals produced by the two techniques (Table 3). Freeze sperm cells entrapped in the freeze matrix were occasionally observed without evident intracellular ice formation (Fig 2D).

**Table 3:** Size and shape descriptors (values are mean ± SE) of crystals assessed by image analysis for both cryopreservation methods.

Size and shape descriptors of crystals		
	Slow freezing	Ultra-rapid freezing
**Area (μm**^**2**^**)**	45.93 ± 3.96^a^	8.98 ± 0.00^b^
**Perimeter (μm)**	29.57 ± 2.05^a^	12.95 ± 0.00^b^
**Aspect ratio**	0.66 ± 0.01^a^	0.65 ± 0.00^b^
**Circularity**	2.18 ± 0.05^b^	2.20 ± 0.00^a^
**Round**	0.56 ± 0.00^a^	0.52 ± 0.00^b^
**Solidity**	0.82 ± 0.00^a^	0.79 ± 0.00^a^

Different lower case letters (a, b) indicate significant differences between crystals found during slow and ultra-rapid freezing states. Shape descriptors (aspect ratio, circularity, round, solidity) are not expressed in units.

### SEM and TEM evaluations

**Fig 3:**
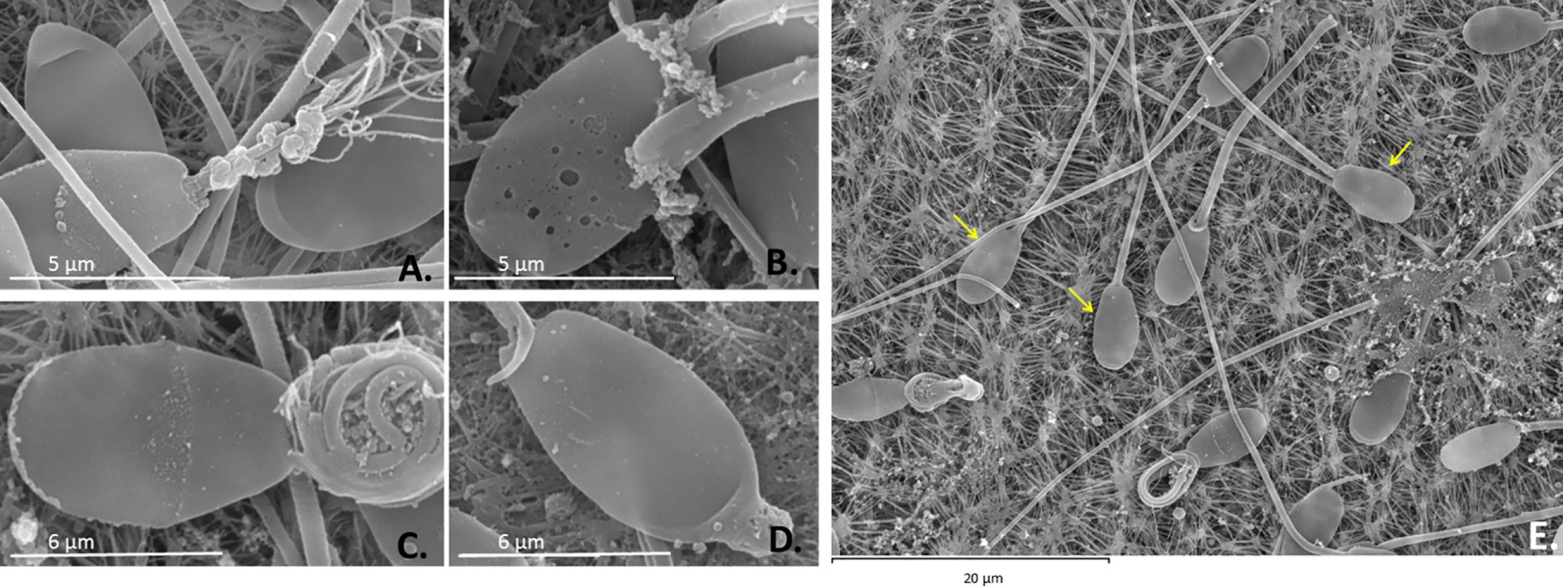
SEM images showing structural damage to thawed ibex sperm after both cryopreservation processes. A and B. Sperm cells after slow cooling with 5% glycerol cryoprotectant. Notice the swollen midpiece damaging the mitochondria (A) and the disruption of the perforated acrosomal membrane (B). C and D. Sperm cells after ultra-rapid cooling with 100 mM sucrose cryoprotectant. Notice the acrosome ridge membrane is wrinkled, the tail rolled, and the midpiece broken. In D, the apical ridge of the acrosome is somewhat swollen (mag. x8500). E. Sperm cells after ultra-rapid cooling. Notice some sperm cells which successfully achieved the cryopreservation process (arrows) (mag. x2500).

With SEM, no significant differences were seen between the cooling methods in terms of damage to the sperm head, acrosome membrane, or mitochondrial structure. In the slow cooled samples, some midpieces appeared to be swollen, causing some mitochondrial damage (Fig 3A), and some regions of the acrosomal membrane showed perforations (Fig 3B). In the ultra-rapid cooled samples some sperms showed a wrinkled (Fig 3C) or swollen (Fig 3D) acrosome ridge membrane, rolled tails, and broken midpieces (Fig 3C).

**Fig 4:**
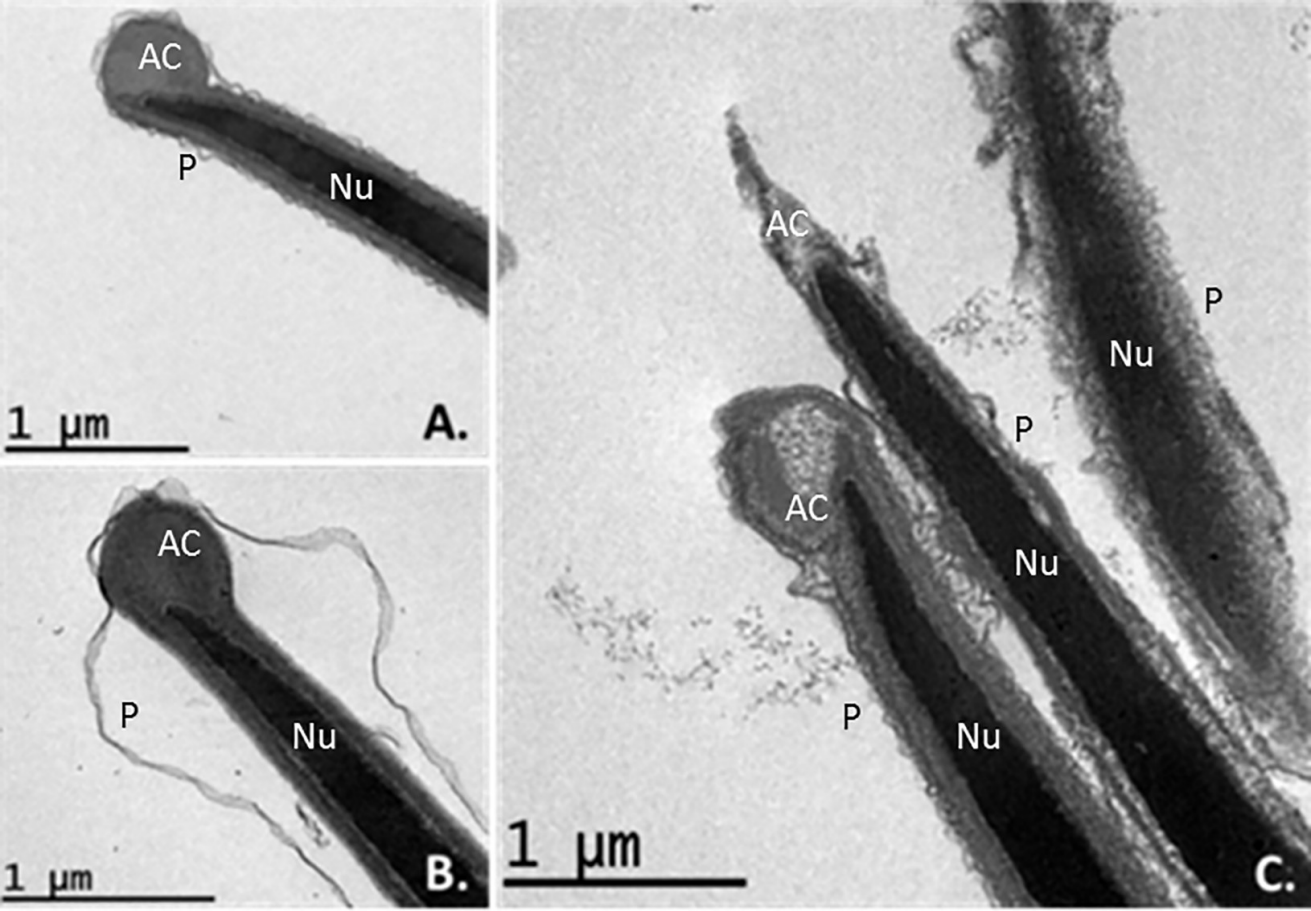
TEM images of osmotically induced pathological changes in transverse and longitudinal sections of thawed ibex sperm heads. Damage to the acrosome membrane is particularly clear irrespective of the cooling rate employed. A and B. Sperm heads after slow-freezing showing an intact acrosome membrane but a shrunken plasma membrane (A) and membrane blebbing (B) (mag. x3000 and x4000). **C.** Sperm head membranes after ultra-rapid freezing. Notice the plasma and acrosome head membranes are reacted, swollen and disintegrated (mag. x3000). Nu = nucleus, AC = acrosome membrane, P = plasma membrane.

Some sperms subjected to slow cooling showed an intact acrosome membrane but a shrunken (Fig 4A) and blebbing plasma membrane (Fig 4B). Some sperms subjected to ultra-rapid cooling showed a swollen or disintegrated acrosome and plasma membranes (Fig 4C). The acrosome membrane also appeared reacted in some cases.

**Fig 5:**
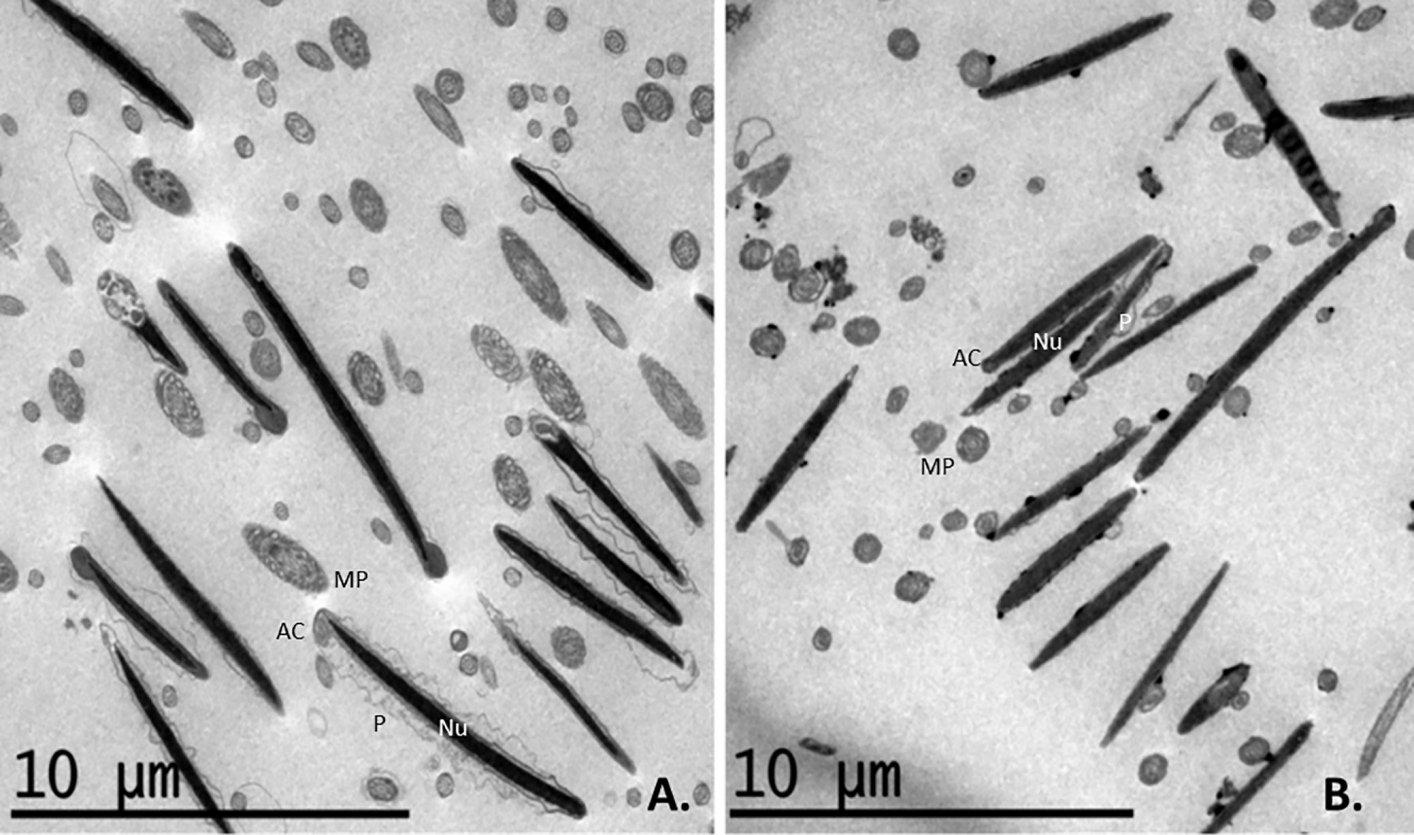
TEM images of transverse and longitudinal sections of thawed ibex sperm following slow and ultra-rapid cooling (mag. in x500). A. Sperm internal structures after slow cooling. Most longitudinal sections of the sperm heads show a swollen plasmalemma in the acrosomal and post-acrosomal region. In transverse sections of the midpiece, the mitochondria show a great deal of vacuolization. B. Sperm internal structures after ultra-rapid cooling. Most of the longitudinal sections of the sperm heads show no plasmalemma since it has disintegrated; some heads and acrosome membranes appear broken. Many mitochondria show vacuolization. The volume of the midpiece is smaller after ultra-rapid cooling (B) than slow cooling (A). Nu = nucleus, AC = acrosome membrane, P = plasma membrane, MP = midpiece.

**Fig 6:**
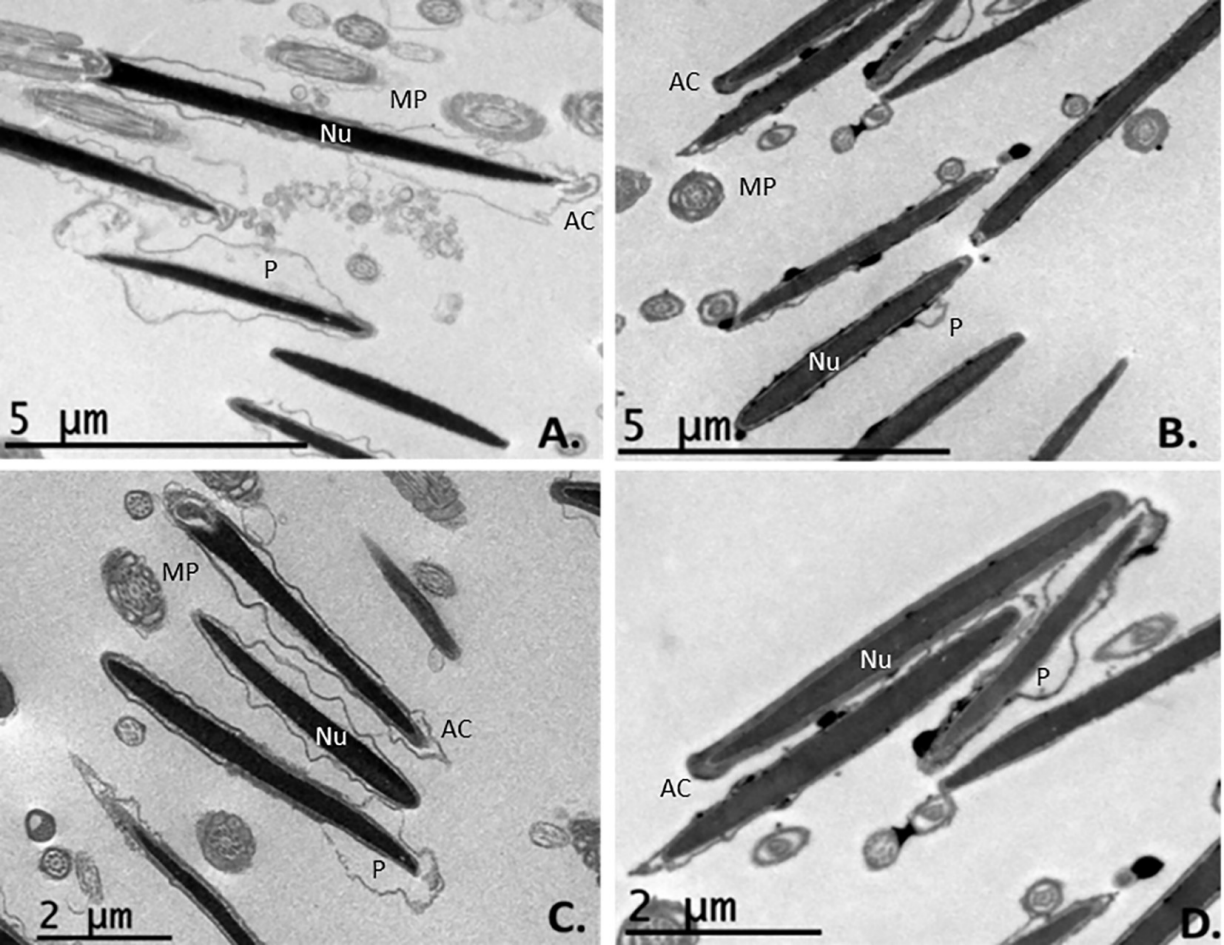
TEM images of transverse and longitudinal sections of thawed ibex sperm following slow and ultra-rapid cooling. A. Sperm structures after slow cooling; transverse sections of sperm heads with some damage to the head membranes and vacuolization of the mitochondria (mag. x1000). B. Sperm structures after ultra-rapid cooling; note the damage to the plasmalemma (sometimes entirely lost) and the vacuolization of the mitochondria (mag. x1000). C. Sperm head and midpiece sections with different plasmalemma damage and mitochondrial vacuolization states after slow cooling (mag. x1200); D. Sperm head and midpiece sections with different degrees of damage to the plasmalemma, membrane detachment along the tail, mitochondrial vacuolization and axoneme destructuring following ultra-rapid cooling (mag. x1500 magnification). The midpiece volume seems smaller after ultra-rapid cooling (B, D) than after slow cooling (A, C). Nu = nucleus, AC = acrosome membrane, P = plasma membrane, MP = midpiece.

**Fig 7:**
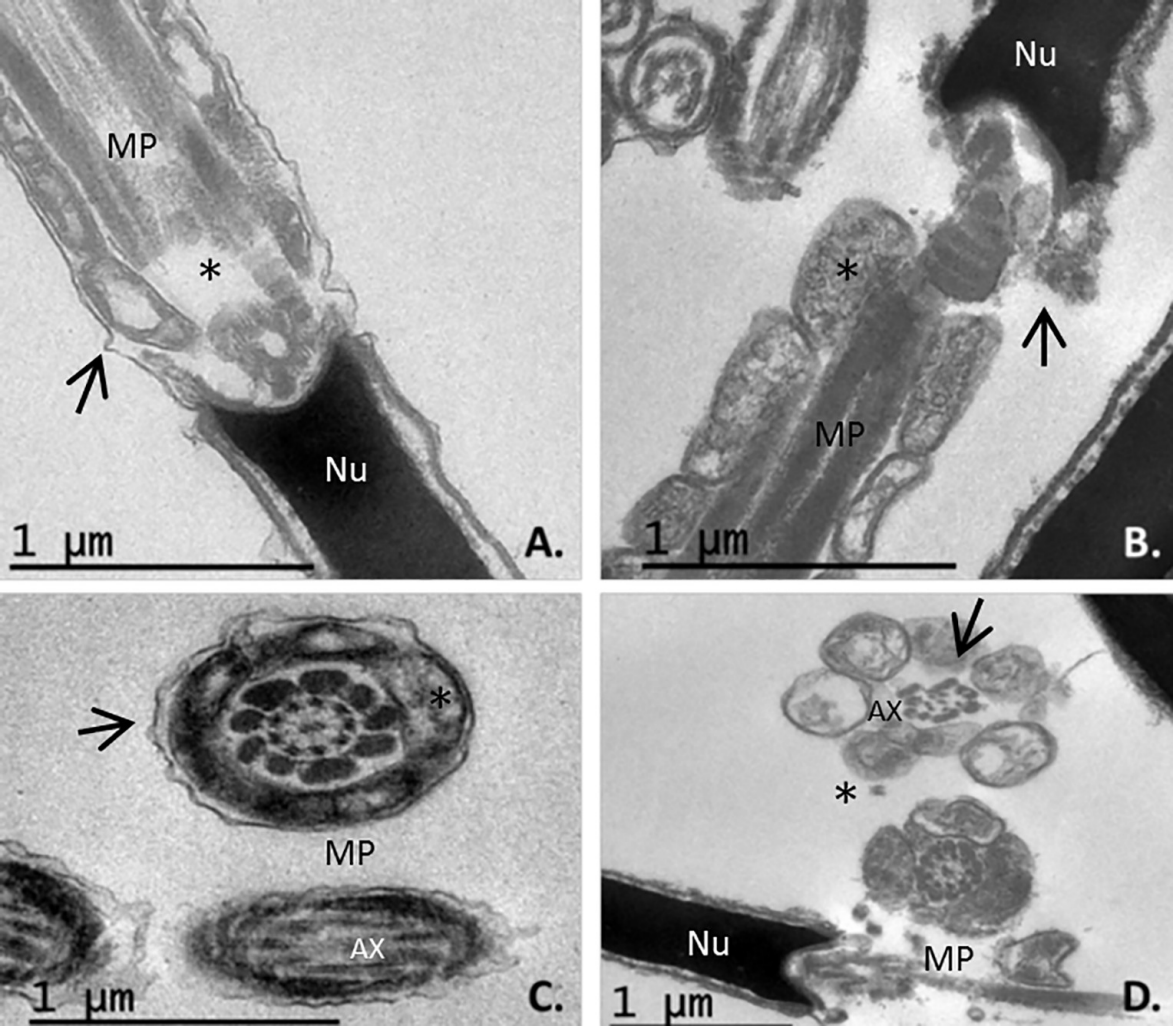
TEM images of osmotically induced pathological changes in transverse and longitudinal sections of the midpiece in thawed ibex sperm after slow and ultra-rapid cooling. A and C. Mitochondria showing vacuolization (asterisks) and detachment of the midpiece plasma membrane (arrows) were seen after slow cooling (mag. x5000). B and D. Sperm cell showing an unstructured midpiece and axoneme (arrows), plus mitochondria showing vacuolization (asterisks), after ultra-rapid cooling (mag. x5000 and x3000). Nu = nucleus, AC = acrosome membrane, P = plasma membrane, MP = midpiece, AX = axoneme.

Under TEM, slow cooling was associated with many sperms showing a swollen plasmalemma in the acrosomal and post-acrosomal head regions in longitudinal section. The same was observed in transverse sections of the tails and midpieces, in which the mitochondria showed notable vacuolization (Figs 5A and 6A and 6C). Ultra-rapid cooling was associated with many sperm heads showing no plasmalemma (it had disintegrated) in longitudinal section (Figs 5B and 6B); some heads and acrosome membranes also appeared broken (Figs 5B and 6B and 6D). Although many mitochondria showed vacuolization, this was less notable than with slow cooling (Figs 5 and 6). In addition, some sperm subjected to ultra-rapid cooling showed membrane detachment along the tail (Fig 6D), and an unstructured axoneme (Figs 6D and 7). Overall, ultra-rapid cooling seemed to be associated with the midpiece being smaller in volume; this was not so common for the slow cooled samples (Figs 5 and 6).

Unstructured axonemes of the tail were observed with both cryopreservation methods. Displacements of microtubules were more usual in sperm samples conventionally frozen (S1 Fig).

## Discussion

This is the first report to assess the ultrastructural damage to ibex sperm cells after cryopreservation. It is suggested by Cryo-SEM method that ultra-rapid cooling failed to induce a stable, glass-like state in the extracellular milieu, but the ice crystal morphology was different to that seen in samples cryopreserved by conventional slow cooling. The sperm cryopreserved by the ultra-rapid cooling method had lower motility values and suffered greater damage to the plasmalemma, acrosome membrane and mitochondrial membrane. Maybe the rapid cooling in the phase prior to ice nucleation could lead to cold shock damage, characterised by a leakage of solutes across to the extracellular milieu, associated with lipid phase transitions in cell membranes [38].

An issue to consider is the possibility that vitrified samples could crystallize during the process of heating to -90ºC, which is made to sublimate free water in the solid state lakes during cryo-SEM procedure. Although estimative methods of glass transition on simple aqueous solution containing carbohydrates could support this circumstance [39], the extender used in the present study also contained Tris, citric acid, and even proteins from the egg yolk. In addition glassy state has a near null sublimation rate, so vitrified solutions do not present enhanced contrast after the etching process [40, 41]. In the same way many reports [42, 43, 44] showed that cryo-SEM allows observations of microstructural features with no alteration due to preparation procedures. All this studies support the use of cryo-SEM to determine the glassy state in cryopreservation process. However, our findings results are only applied to this specific cryopreservation method used, with its own characteristics (e.g. pellet volume, diluent composition, etc.) that may differ from others.

The present results did not reveal evidence of any intracellular ice in the head sperm entrapped in the freeze matrix with both ultra-rapid and slow cooling rates. This agree with other reports using different cooling rates in which examined sperm do not contain intracellular ice i.e. vitrification of the intracellular compartment always occur [9, 45, 46]. However, small homogeneous ice crystals may not be observed by this SEM technique, and thus only the absence of large ice crystals can be shown. The present study shows that there are not clear evidences for intracellular ice formation within the sperm head, unlike larger cells (e.g. oocytes and embryos). Not all studies support the idea that intracellular ice forms even when using slow cooling rates (which theoretically should be more likely) [45, 47]. Thus, intracellular vitrification (in which no crystals form) may be quite easy to achieve in sperm cells [12] even by conventional slow cooling [9], a likely consequence of their small size and high soluble macromolecule content. Certainly, it is well known that vitrification depends on the interaction between the cooling rate, the viscosity and volume of the solution [20].

It has also long been assumed that few crystallization nuclei form in the extracellular milieu during the ultra-rapid cooling of small volumes, and that any that do are too small to damage cells or cause any substantial shrinkage [12]. However, the present findings do not support this idea; the ultra-rapid freezing rate did not prevent the formation of ice crystals in the extracellular milieu. In the samples thus treated, severe membrane damages was observed, and the changes recorded in the sperm head dimensions suggest the cells suffered shrinkage and then swelling over the freezing-thawing process. However, these findings should be understood with caution since there is scant information on this technique, and the volume used in the present study (50 μL) was greater than that used in other studies to develop kinetic vitrification (20 μL) [48]. Differences in the cooling rate may also help explain the differences seen in the shape descriptors and size of the crystals formed during ultra-rapid and slow cooling [49]. Extender composition might also affect the crystallization process. Sucrose is thought to slow down ice nucleation [49, 50]; the idea that it may influence the shape of any ice crystals formed cannot, therefore, be ruled out. After ultra-rapid cooling, the crystals were smaller and more stretchmarked than those observed after slow cooling. This pattern of ice formation in ultra-rapid method might favour a direct contact of ice with cells, producing a greater damage.

Differences in the characteristics of the ice crystals in the extracellular milieu produced by the tested methods might lead to differences in the abundance of pockets of high solute concentration, subjecting the cells to different levels of osmotic stress. It is well known that cryopreservation reduces sperm in motility and viability, and that it modifies cell morphometric variables. Such damage might be related to the osmotic stresses resulting from the water–solute interactions that arise because of crystallization [51]. As dissolved salts are excluded from the forming ice, they become concentrated between ice crystals forming pockets of high solute concentration. These would draw out of cells, leading to their shrinkage, and eventually bursting of the membrane [52]. Although extracellular ice seems a primary cause of sperm damage during the cryopreservation process [23], some intracellular ice formation cannot be ruled out, in cell compartments other than the head, as sperm midpiece and tails [53, 54]. Indeed, our findings showing the displacements of microtubules of tail (S1 Fig) might be produced by ice crystals. Reduction in sperm motility might be related to damage caused to the mitochondrial membranes [55], which was more severe in the present ultra-rapidly cooled sperms (as shown by MITO-fluorescence analysis). Some sperm morph-structural variables, such as the status of the plasma membrane, acrosome membrane and mitochondrial membrane are related directly with the functionality of sperm cells. The last correlates with the sperm motility, but the others two are not necessary for this parameter, being related with others fertility functions. Sperm cells with damaged acrosome and/or plasmalema are non-functional and thus are not considered to be successfully preserved, even if they have intact mitochondria and are able to move. The term of “motile sperm” includes total progressive and non-progressive sperm. Certainly, the percentage of sperm with intact plasmalema, acrosome and mitochondrial membranes (IP-IA-IM, 14%) were lower than the percentage of motile sperm (23%) in samples by slow cooling. This fact reveals that sperm with the plasmalema damaged may show kinetic activity if the mitochondria still remains unaltered. Even though the percentage of intact mitochondrial membranes was less than percentage of motile sperm for slow conventional freezing, the results are in agreement with other study about bovine sperm cryopreservation [35]. The damage caused to the mitochondrial membrane during slow cooling and later thawing, which affected about 83% of the sperm cells, was probably caused by swelling (Fig 3A) and vacuolization (Figs 5A and 6A and 6C). These quantitative changes were accompanied by differences in the ultrastructural changes suffered by the mitochondria (as shown by SEM and TEM). The ultra-rapid cooling technique affected 96% of sperm mitochondria (as shown by fluorescence), perhaps caused by mitochondrial vacuolization (this value fell to 83% with slow cooling). TEM also revealed the midpiece to have a smaller volume after ultra-rapid cooling than after slow cooling, suggesting an inability to return to initial volumes after osmotic shock, perhaps due to irreversible damage to the plasma membrane [10]. This would explain the strong reduction in motility when ultra-rapid cooling is employed [19].

The SEM results show that, with both freezing methods, some sperm suffer a fractured plasmalemma and a disrupted acrosome membrane. Similar damage has been encountered in sperm samples from other species after freezing-thawing, e.g. in goats [56], cattle [57] and the blue fox [58]. Krogenaes et al. [57] report that, in TEM images of frozen-thawed bull sperm, the plasmalemma appeared swollen and detached from the sperm head. Such damage, along with vacuolization of the mitochondria, might be associated with osmotic shock [10] suffered during freezing-thawing. Ultrastructural changes in the acrosomes and mitochondria were described as similar to those seen in bovine and ovine sperm frozen by conventional techniques [59, 60]. González-Fernández et al. [10] also suggest that all these kinds of ultrastructural alteration in equine sperm are likely produced by osmotic changes. In addition, they report that under hypotonic conditions (75 mOsm), TEM images showed the sperm cell plasmalemma to be swollen and detached from the sperm head, and mitochondrial to be volume increased; the mitochondrial volume decreased under hypertonic conditions. Similar osmotic changes might therefore be expected during freezing-thawing.

In conclusion, ultra-rapid cooling of ibex sperm by spheres is a promising and useful alternative to conventional freezing methods, mainly when sperm cryopreservation is performed in wild species under field conditions with lack of sophisticated equipment. This technique did not prevent the production of extracellular ice; the extracellular milieu did not achieve a truly vitrified state. Although ultra-rapid cooling simplifies the process of cryopreservation and it has been successfully used in other wild species [28], it causes severe damage to sperm cell membranes, and thus effort should be focused to investigate new additives-cryoprotectants and to optimize the volume of pellets for avoiding the extracellular ice formation.

## Supporting information

S1 Fig TEM images showing structural damage to ibex sperm axonemes. Notice the displacements of microtubules (arrows) and vacuolization of mitochondria (asterisks).
(TIF)
