# Supplementary figures and images for: Ultra-rapid cooling of ibex sperm by spheres method does not induce a vitreous extracellular state and increases the membrane damages
Source: PLoS One. 2020 Jan 24;15(1):e0227946. doi: 10.1371/journal.pone.0227946 (PMC6980613; doi:10.1371/journal.pone.0227946)

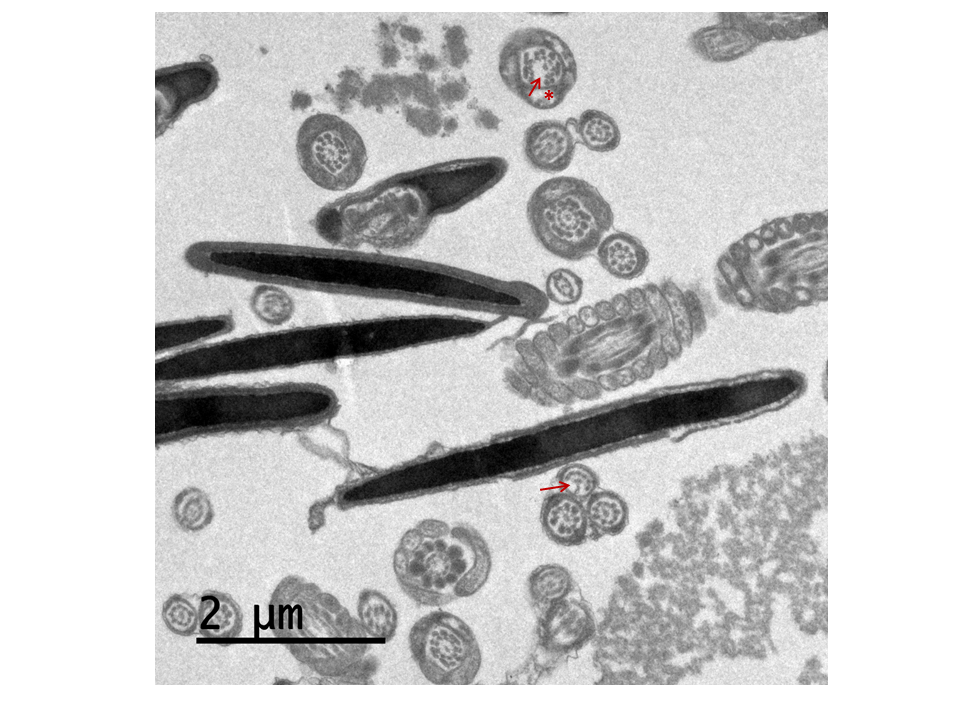

Supplement: S1 Fig — Notice the displacements of microtubules (arrows) and vacuolization of mitochondria (asterisks). (TIF) [file pone.0227946.s001.tif]
